# *KRAS4A* and* KRAS4B* in liquid biopsy of metastatic lung adenocarcinoma patients treated with Pembrolizumab or chemotherapy plus Pembrolizumab

**DOI:** 10.1038/s41598-023-48304-0

**Published:** 2023-11-29

**Authors:** Rita Chiari, Silvia Palladino, Rita Emili, Mariagrazia De Lisa, Donatella Sarti, Vincenzo Catalano, Mauro Magnani, Francesco Graziano, Annamaria Ruzzo

**Affiliations:** 1 Oncology Unit, AST1 Pesaro e Urbino, Stabilimento di Muraglia - Via Lombroso 1, 61122 Pesaro, Italy; 2 Department of Biomolecular Sciences, University of Urbino “Carlo Bo”, Via Arco d’Augusto, 2, 61032 Fano, Italy; 3 Oncology Unit, AST1 Pesaro e Urbino, Urbino, Italy; 4 Oncology Unit, AST1 Pesaro e Urbino, Fano, Italy; 5 Department of Biomolecular Sciences, University of Urbino “Carlo Bo”, Urbino, Italy

**Keywords:** Gene expression analysis, Lung cancer, Non-small-cell lung cancer

## Abstract

*KRAS* is involved in the stability and expression of PD-L1*.* We investigated the expression of circulating mRNA (cmRNA) of *KRAS4A* and *KRAS4B* and the possible impact on progression-free survival (PFS) of patients with metastatic lung adenocarcinoma treated with immunotherapy. Patients without driver mutations undergoing Pembrolizumab (P) or P plus chemotherapy (PC) were prospectively accrued for liquid biopsy analysis of *KRAS4A*, *KRAS4B,* and *PD-L1* cmRNA. Both *KRAS* isoforms were also studied for association with *PD-L1* cmRNA. Of 56 patients, 28 received P and 28 PC. Patients with high levels of both *KRAS* isoforms showed significantly better PFS. The median PFS for *KRAS4A* was 29 months (95% CI 22–29 months) and *KRAS4B* 24 months (95% CI 13–29 months), respectively. The median PFS of patients with low levels of both isoforms was 12 months (95% CI 6–15 months for *KRAS4A* and 95% CI 5–20 months for *KRAS4B*). High *KRAS4A* retained a significant positive association with PFS in the multivariate model. An exploratory analysis in treatment subgroups found a positive association between high *KRAS4A* and *KRAS4B* with PFS in patients treated with P. *PD-L1* cmRNA was significantly higher in patients with high *KRAS* isoforms levels and this effect was pronounced for high *KRAS4A* carriers. *KRAS4A* deserves further investigation as a potential marker for defining patients who may benefit the most from immune checkpoint inhibitors therapy and improving personalized cancer immunotherapeutic strategies.

## Introduction

Immune checkpoint inhibitors (ICI) targeting either programmed cell death protein 1 (PD-1) or programmed cell death ligand 1 (PD-L1) have become routinely part of the clinical approach for the management of Non-Small Cell Lung Cancer (NSCLC)^1^. Factors that affect the choice of treatments in NSCLC that lack a driver mutation include the level of PD-L1 expression on cancer cells, the extent of the disease and the tumor's histological characteristics. Specifically, patients with metastatic NSCLC and PD-L1 expression ≥ 50% are typically offered monotherapy with the anti-PD-1 antibody Pembrolizumab. For patients with PD-L1 expression < 50%, the combination of platinum-doublet chemotherapy and Pembrolizumab is the standard^1^. These treatment strategies have significantly improved the overall management of metastatic NSCLC with the capability to induce long-term responses and prolonged survival times in settings often refractory to all other treatments. Unfortunately, a relevant portion of patients does not respond to these treatments and/or they do not gain meaningful improvements in survival times^2^. To break through the limits of clinical effects, it is critical to make a profound understanding of the underlying mechanisms of sensitivity to expand the benefit of immunotherapy^2^.

*KRAS* mutations occur in 20–40% of lung adenocarcinomas, a prevalence that is higher in Western than in Asian populations (26 vs. 11%) and higher in smokers than non-smokers (30 vs. 10%)^3^. Retrospective analyses in clinical trials of ICI in metastatic lung cancer patients described a possible association between *KRAS* mutations and improved clinical outcomes^3^. In fact, the activity of Kras along the receptor tyrosine kinase (RTK)-Ras-MAPK pathway has been linked to immunomodulation^4^. Ras signaling can upregulate PD-L1 expression on tumor cells by stabilizing *PD-L1* mRNA^5,6^ and increasing protein levels of PD-L1 with induction of the MAPK, PI3K and Akt pathways^7–9^.

In recent years, translational studies have been conducted in Asian and Western populations. In a retrospective analysis of a Chinese population of patients with *KRAS*-mutant advanced NSCLC, immunotherapy-based regimens achieved longer overall survival (OS) than chemotherapy-based regimens, which was independent of first or second-line setting, as well as of *KRAS* mutational subtypes^10^. The Food and Drug Administration (FDA) pooled data from 12 registrational clinical trials investigating ICI with or without chemotherapy, showing that patients with *KRAS*-mutated NSCLC derived the most significant benefit from the combination of chemotherapy-ICI as compared to ICI or chemotherapy alone^11^.

Therefore, pre-clinical and clinical data have propelled research in *KRAS* analyses for defining populations of metastatic cancer patients who may benefit the most from current immunotherapy. A more precise evaluation of the role of *KRAS* for predictive/prognostic purposes should consider the increasing evidence on the role of *KRAS4A* and *KRAS4B* isoforms^12^. The precursor mRNA (pre-mRNA) of *KRAS* is alternatively spliced to give rise to two products, Kras4A and Kras4B. The number of amino acids and molecular weight of Kras4A and Kras4B are almost identical. However, the last 24 and 25 amino acids on their C-terminal regions are coded by different exons and are, therefore, significantly different. The different C-terminal results in the aforementioned difference in post-translational modification, namely, the palmitoylation of Kras4A but not Kras4B^13^. Kras4A-specific interacting proteins are involved in mitosis, DNA damage, ion transport and apoptosis^14^. The wild-type Kras4B showed anti-apoptotic activity in animal models, while wild-type Kras4A showed pro-apoptotic activity^15^. It was proven in experimental carcinogenesis models that both homozygous and heterozygous *KRAS4A* knockout animals produced larger colonic adenocarcinomas with shorter survivor, with wild-type *KRAS4A* having a tumor suppressive effect^16^. Adenomas not expressing *KRAS4A* had significantly increased cell proliferation and significantly decreased apoptotic activity with evidence of activation of MAPK and Akt pathways^16^. Notably, in experimental models, the Kras4A-RAF1 interaction led to increased RAF-MEK-ERK signaling^14^, which in turn contributed to PD-L1 stabilization^17^. Due to the reversibility of palmitoylation, Kras4A exists in bimodal signaling states which may occur under different oncogenic cell/tissue conditions. The de-palmitoylated Kras4A is co-localized with hexokinase 1 (HK1) at the outer mitochondrial membrane. In experimental models, Kras4A was found to regulate HK1^18^, which catalyzes the first step of glucose metabolism with glucose phosphorylation to form glucose-6-phosphate. According to experimental and pre-clinical studies, the enhanced glycolytic flux due to the Kras4A*-*HK1 interaction^18^ may sustain an immune-stimulatory tumor microenvironment^19^. Moreover, glycolytic activity may enhance PD-L1 expression on tumor cells and thus promote anti-PD-1/PD-L1 immunotherapy response^19^. Emerging work has confirmed the non-overlapping functions of the splice variants. Also, the observation that Kras4B but not Kras4A is localized on lysosomes may explain differential protein–protein interactions^20^. The findings that both isoforms are widely expressed in cancers with heterogeneity across tumor tissues and regardless of the mutational *KRAS* status^21–23^ make them worthy of translational studies addressing their role in cancer progression and resistance to current treatment options, including immunotherapy.

According to this background and considering the feasibility of mRNA quantification for both *KRAS* isoforms and *PD-L1* in liquid biopsy^24^, we planned a prospective study in metastatic lung cancer patients who were candidates to first-line systemic therapy with Pembrolizumab alone or in combination with standard chemotherapy. The cmRNA expression levels of *KRAS4A* and *KRAS4B* results were analyzed and associated with PFS and cmRNA expression levels of *PD-L1*.

## Results

### Study population

Between August 2021 and December 2022, sixty patients were enrolled. Four patients were not assessable: one patient due to technical failure of mRNA analysis, one patient did not receive Pembrolizumab for medical reasons and two patients were staged with locally advanced, non-metastatic disease. The characteristics of the 56 fully assessable patients are listed in Table 1. There were 28 patients treated with Pembrolizumab and 28 patients treated with Pembrolizumab plus chemotherapy. At the time of the analysis (September 2023), 33 patients had progression (59%), and the remaining 23 (41%) were censored observations. In the whole group, the median PFS time was 15.5 months (95% CI 12–24 months).

**Table 1:** Characteristics of the 56 patients and distribution according to treatment.

Variable	Total	Number of patients (%)		*p-*value
		Pembrolizumab/Chemotherapy	Pembrolizumab	
Age				
> 65 years	37 (66.1)	18 (64.2)	19 (67.8)	1
< 65 years	19 (33.9)	10 (35.8)	9 (32.2)	
Gender				
Male	41 (73.2)	19 (67.8)	22 (78.5)	0.5
Female	15 (26.8)	9 (33.2)	6 (21.5)	
*KRAS* status				
Wild-type	43 (76.7)	22 (78.5)	21 (75.0)	1
Mutated	13 (23.3)	6 (21.5)	7 (25.0)	
Number or metastatic sites				
1–2	36 (64.2)	19 (67.8)	17 (60.7)	0.7
> 2	20 (35.8)	9 (33.2)	11 (39.3)	
CNS involvement				
Positive	16 (28.5)	7 (25.0)	9 (31.1)	0.7
Negative	40 (71.5)	21(75.0)	19 (68.9)	
ECOG PS				
0–1	46 (79.3)	21 (75.0)	25 (89.2)	0.3
2	10 (20.7)	7 (25.0)	3 (10.8)	
*KRAS4A* expression*				
High	28 (50.0)	12 (42.9)	16 (57.1)	0.4
Low	28 (50.0)	16 (57.1)	12 (42.9)	
*KRAS4B* expression*				
High	31 (55.3)	14 (50.0)	17 (60.7)	0.6
Low	25 (44.7)	14 (50.0)	11 (39.3)	

*High and Low expression of the two *KRAS* isoforms according to the Youden index (see text).

*ECOG PS* Eastern Cooperative Group Performance Status, *CNS* central nervous system.

Mean Cy_0_-values for *KRAS4A* and *KRAS4B* cmRNA expression levels were 30.33 (95% CI 29.96–30.70) and 29.47 (95% CI 29.02–29.92), respectively. Correlation analysis showed that Cy_0_-values for *KRAS4A* and *KRAS4B* are highly correlated (0.99 Pearson correlation coefficient). Mean Cy_0_-values for *PD-L1* cmRNA was 32.90 (95% CI 32.67–33.13), the Pearson correlation coefficient between *KRAS* isoforms and the *PD-L1* Cy_0_ was 0.35.

A random sample of 14 patients (25%) underwent a second liquid biopsy after 12 weeks at the time of first re-staging of disease (T1). We found comparable results between baseline (T0) and T1 cmRNA values. In detail, mean Cy_0_-values for *KRAS4A* cmRNA at T0 and T1 were 31.38 (95% CI 30.61–32.14) and 31.01 (95%CI 29.98–32.02), respectively. Mean Cy_0_-values for *KRAS4B* cmRNA at T0 and T1 were 30.71 (95% CI 29.67–31.64) and 30.23 (95%CI 28.87–31.58), respectively. Mean Cy_0_-values for *PD-L1* cmRNA at T0 and T1 were 33.43 (95% CI 33.04–33.82) and 33.05 (95%CI 32.41–33.69), respectively.

The median PFS time in the 28 patients treated with Pembrolizumab was 24 months (95% CI 15–29 months). Mean Cy_0_-values for *KRAS4A* and *KRAS4B* cmRNA expression levels were 30.27 (95% CI 29.71–30.84) and 29.42 (95% CI 28.73–30.10). In the 28 patients treated with Pembrolizumab plus chemotherapy, the median PFS time was 12.5 months (95% CI 6–23 months). Mean Cy_0_-values for *KRAS4A* and *KRAS4B* mRNA expression levels were 30.39 (95% CI 29.87–30.90) and 29.52 (95% CI 28.88–30.15).

### PFS and KRAS isoforms expression

In the analysis of the *KRAS4A* isoform in the 56 patients, based on ROC (area under the curve, AUC_4A-plasma_ = 0.731, 95% CI 0.596–0.841), the Youden index (the maximum product of sensitivity and specificity observed) achieved 73% sensitivity and 74% specificity when the Cy_0_-values > 29.72 was used as the cut-off for Kaplan–Meier plots. In the analysis of the *KRAS4B* isoform in the 56 patients, based on ROC (area under the curve, AUC_4B-plasma_ = 0.697, 95% CI 0.559–0.812), the Youden index (the maximum product of sensitivity and specificity observed) achieved 62% sensitivity and 74% specificity when the Cy_0_-values > 28.74 was used as the cut-off for Kaplan–Meier plots.

**Figure 1:**
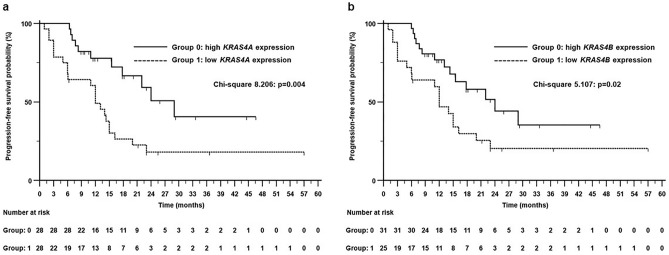
Kaplan–Meier Progression-Free survival curves with the distribution of the 56 patients according to high (H) and low (L) expression levels of *KRAS4A* cmRNA (**a**) and *KRAS4B* cmRNA (**b**).

As shown in Fig. 1, in the Kaplan–Meier plots, there was a statistically significant difference in PFS times between patients with high and low expression of the *KRAS4A* and the *KRAS4B* cmRNA isoforms (Fig. 1a and b, respectively). In both analyses, patients classified with high expression showed better PFS than patients classified with low expression with a more pronounced effect for the *KRAS4A* isoform. The median PFS times of patients with high *KRAS4A* and high *KRAS4B* expressions were 29 months (95% CI 22–29 months) and 24 months (95% CI 13–29 months), respectively. Median PFS of patients with low *KRAS4A* and low *KRAS4B* cmRNA expression were 12 months (95% CI 6–15 months) and 12 months (95% CI 5–20 months), respectively.

Considering the results of the log-rank test analyses for PFS and the results of the Pearson correlation that showed a high correlation between the expression levels of the two *KRAS* isoforms, only the expression levels of the *KRAS4A* were included in the multivariate model (Table 2). The analysis also considered all available clinical features and the *PD-L1* cmRNA expression. The *KRAS4A* expression retained a significant association with PFS.

**Table 2:** Results of the multivariate model analysis for progression-free survival.

Variable	HR (95% confidence interval)	*p-*value
Age		
> 65 years versus < 65 years	1.02 (0.40–2.60)	0.9
Gender		
Male versus Female	1.26 (0.48–3.08)	0.6
*KRAS* status		
Mutated versus wild-type	1.21 (0.40–2.60)	0.3
Number of metastatic sites		
> 2 versus 1–2	1.05 (0.41–2.70)	0.9
ECOG-PS		
2 versus 0–1	1.54 (0.61–3.76)	0.3
Treatment		
PC versus P	1.79 (0.80–3.72)	0.1
CNS metastasis		
Absent versus Present	0.67 (0.28–1.61)	0.3
*KRAS4A* isoform expression*		
High versus Low	0.31 (0.11–0.87)	0.02
PD-L1 expression*		
High versus Low	0.69 (0.25–1.84)	0.3

*High and low expression of the the *KRAS4A* isoform and *PD-L1* according to the Youden index (see text).

*HR* hazard ratio, ECOG-PS, Eastern Cooperative Group Performance Status, *P* pembrolizumab, *PC* pembrolizumab/chemotherapy, *CNS* central nervous system.

**Figure 2:**
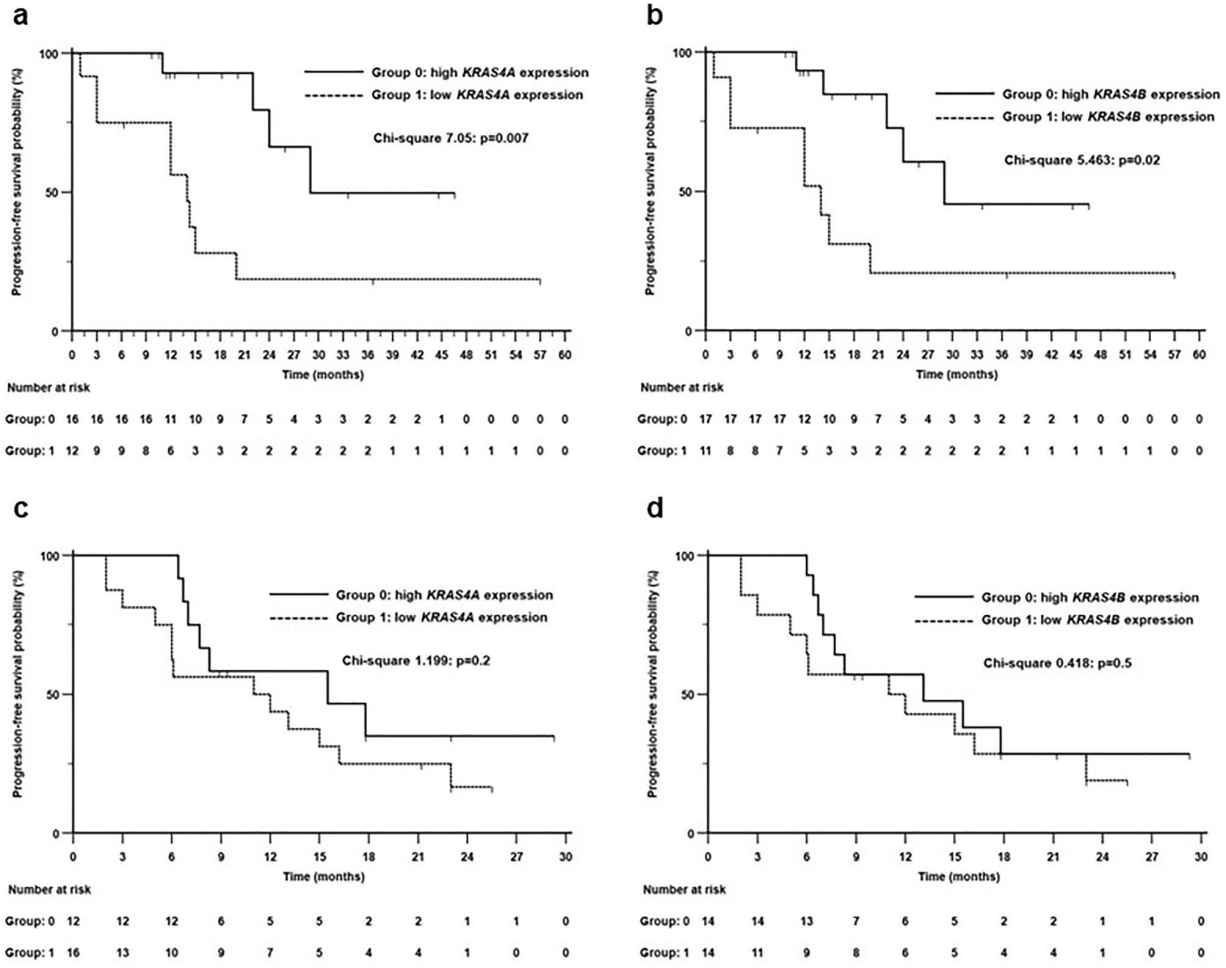
Kaplan–Meier Progression-Free survival curves in the 28 patients treated with Pembrolizumab (**a** and **b**) and in the 28 patients treated with Pembrolizumab plus chemotherapy (**c** and **d**).

An exploratory analysis was performed according to treatment groups (Fig. 2). In the 28 patients treated with Pembrolizumab (PD-L1 expression ≥ 50%), patients with high *KRAS4A* and high *KRAS4B* expression (Fig. 2a and b, respectively) showed significantly better PFS time than patients with low *KRAS4A* and low *KRAS4B* cmRNA expression. In the 28 patients treated with Pembrolizumab plus chemotherapy (PD-L1 expression < 50%), none of the two isoforms (Fig. 2c and d, respectively) showed a significant split of PFS curves between high and low expression groups.

As far as *PD-L1* cmRNA expression is concerned, high levels (Youden index of Cy_0_-values > 32.87) were associated with reduced risk of progression (0.69 Hazard Ratio, 034–1.37 95% confidence intervals), but without achieving statistical significance.

### Objective responses and isoforms expression

The expression of the *KRAS4A* and *KRAS4B* cmRNA isoforms was analyzed in the context of objective responses attained with immunotherapy alone or combined with chemotherapy (Table 3). There was an overall trend for higher response rates (complete plus partial responses) in high *KRAS4A* and *KRAS4B* cmRNA expression groups. Notably, a statistically significant difference was achieved in the analysis of patients treated with Pembrolizumab alone, who were selected for single-agent immunotherapy because of PD-L1 > 50% in tumor tissues.

**Table 3:** Association analysis between circulating *PD-L1* cmRNA levels and *KRAS4A* cmRNA, *KRAS4B* cmRNA, *KRAS* mutational status and PD-L1 immunohistochemical expression.

Variable	Number of patients (%)	*PD-L1 *cmRNA levels Arithmetic mean (Cy_0_* value)	*PD-L1 *cmRNA levels 95% CI for the mean (Cy_0_* value)	Two-tailed probability
High *KRAS4A* cmRNA	28 (50)	32.56	32.35–32.77	*p* = 0.001
Low *KRAS4A* cmRNA	28 (50)	33.29	32.90–33.67	
High *KRAS4A* cmRNA	31 (55)	32.57	32.38–32.78	*p* = 0.004
Low *KRAS4A* cmRNA	25 (45)	33.36	32.94–33.77	
*KRAS* wild-type	43 (76)	32.93	32.63–33.21	*p* = 0.9
*KRAS* mutated	13 (34)	32.92	32.58–33.26	
PD-L1 ≥ 50% IHC	28 (50)	32.97	32.69–33.25	*p* = 0.6
PD-L1 < 50% IHC	28 (50)	32.80	32.49–33.26	

*Cy_0_-value is inversely correlated with the amount of the target in the assay. Therefore, a higher cmRNA level corresponds to a smaller Cy_0_-value.

*CI* confidence interval, *IHC* immunohistochemistry.

### Expression analysis

First, the high and low cmRNA levels of *PD-L1*, *KRAS4A* and *KRAS4B* were analyzed for association with clinical features of the study population, including the metastatic pattern (i.e., the presence of brain lesions) and *KRAS* mutational status. However, no significant association was observed (data not shown).

Then, the expression of the *KRAS4A* and *KRAS4B* cmRNA isoforms, together with *KRAS* mutational status and PD-L1 tumor expression by immunohistochemistry (IHC) were evaluated for their association with *PD-L1* cmRNA level. Table 4 shows the results of this analysis. The mean cmRNA *PD-L1* Cy_0_-value was significantly higher in the high expression group of both *KRAS4A* and *KRAS4B* isoforms. The *KRAS* mutational status and PD-L1 tumor expression by IHC did not seem to correlate with *PD-L1* cmRNA Cy_0_-value.

**Table 4:** Distribution of objective responses in the 56 patients according to *KRAS4A* and *KRAS4B* cmRNA expression and treatment groups.

Variable^§^	Number of patients (%)		*p-*values
	Responders*	Non-responders**	
All patients			
*KRAS4A*			
High	22 (59.5)	6 (31.5)	0.08
Low	15 (40.5)	13 (68.5)	
*KRAS4B*			
High	24 (64.8)	7 (36.8)	0.05
Low	13 (35.2)	12 (63.2)	
Chemotherapy/Pembrolizumab			
*KRAS4A*			
High	8 (47.0)	4 (36.6)	0.5
Low	9 (53.0)	7 (63.4)	
*KRAS4B*			
High	10 (58.8)	4 (36.6)	0.2
Low	7 (41.2)	7 (63.4)	
Pembrolizumab			
*KRAS4A*			
High	14 (70.0)	2 (25.0)	0.04
Low	6 (30.0)	6 (75.0)	
*KRAS4B*			
High	14 (70.0)	3 (37.5)	0.1
Low	6 (30.0)	5 (62.5)	

*Patients with complete or partial response.

**Patients with stable disease or disease progression.

^§^High and Low expression of the two *KRAS* isoforms according to the Youden index (see text).

## Discussion

To the best of our knowledge, this is the first report focusing on *KRAS* isoforms cmRNA with emphasis on clinical outcomes in patients with metastatic lung adenocarcinoma treated with an ICI. The main findings are the association between high levels of *KRAS4A* and *KRAS4B* cmRNA high levels and high levels of *PD-L1* cmRNA and the correlation between high levels of *KRAS4A* cmRNA and improved PFS. However, in the multivariate model, high levels of *KRAS4B* cmRNA showed a positive effect on PFS*,* but the association of this isoform did not achieve statistical significance. In the exploratory analysis according to treatment subgroups, patients with high tumor PD-L1 expression (≥ 50%) by IHC who received single-agent pembrolizumab seemed to derive the most significant PFS benefit in the presence of high *KRAS4A* cmRNA levels. Moreover, the responses analysis seems to parallel these findings with improved objective response rates in patients with high *KRAS4A* cmRNA, especially in patients treated with single-agent Pembrolizumab.

The results of our study confirm previous pre-clinical and translational studies indicating that Ras signaling can up-regulate PD-L1^5–9^, which in turn may improve the therapeutic effect of an ICI^20^. In fact, high *KRAS4A* and *KRAS4B* levels were significantly associated with *PD-L1* cmRNA levels. Notably, the same association was not detected when *KRAS4A* and *KRAS4B* quantification was correlated with tumor PD-L1 expression by IHC. This finding could be explained on the basis of the “dynamic” of PD-L1 expression in tumor tissues. To this regard, several studies have demonstrated that PD-L1 expression is highly heterogeneous between primary and metastatic lesions^20^. Hwang et al.^25^ found a significantly higher PD-L1 expression in metastatic lung cancer specimens than in the corresponding primary tumor. Moreover, cellular intrinsic factors may regulate PD-L1 expression over time^20^. In fact, Lacour et al.^26^ showed a significant increase in PD-L1 expression at tumor recurrence in NSCLC.

Although high *KRAS4A* and high *KRAS4B* cmRNA levels were comparable to high *PD-L1* cmRNA levels, *KRAS4A* only displayed an independent association with PFS. Due to the relatively small sample size, it cannot be ruled out that also *KRAS4B* may achieve a statistically significant association with survival outcomes. However, in the only two available translational studies on *KRAS* isoforms, Yang et al.^27^ and Abubaker et al.^28^ found an association between *KRAS4A* abundance measures (high expression and proportion) and favorable OS of patients with lung adenocarcinoma and colorectal carcinoma. Likely, differences in biological effects between the two isoforms and peculiar properties of *KRAS4A* should be taken into account.

The exploratory analysis by treatment subgroups suggests that the positive effect on PFS of high *KRAS4A* quantification seems dominant in patients with high (≥ 50%) PD-L1 tumor expression and therefore treated with single-agent Pembrolizumab. Conversely, the impact of high *KRAS4A* levels seems weakened in patients treated with chemotherapy plus Pembrolizumab because of low (< 50%) PD-L1 expression. Notably, in the present study, among the 28 patients treated with chemotherapy plus Pembrolizumab, 20 patients (71.5%) had PD-L1 positivity between 1 and 5% (data not shown). These data suggest firstly that the effect of *KRAS4A* on *PD-L1* expression or stabilization may be a mechanism that impacts the treatment outcomes of immunotherapy^5–9^. Secondly, the interplay between *KRAS4A* and *PD-L1* may be additive and the magnitude of the clinical effect increases according to the intrinsic *PD-L1* levels in the tumor microenvironment. Overall, these signals would suggest that further investigation on *KRAS4A* expression levels as predictive marker could be useful in patients with high *PD-L1* expression.

Obviously, these results should be looked at with caution because of the limited sample size. Furthermore, they deserve validation in additional studies. Also, additional experimental studies should corroborate the hypothesis that links *KRAS* isoforms to the induction of the Ras pathway and finally to PD-L1 stimulation or stabilization. Another limitation of the study is the lack of information about other mutational or expression analysis in tumor tissue (i.e. *TP53,* MAPK plus other effectors of the *KRAS* cascade). However, it should be considered that small biopsies are obtained in the diagnostic evaluation of metastatic lung cancer. A histopathologic sample is usually collected from larger gauge needle biopsies (also referred to as *core biopsies*), such as those commonly performed during computed tomography-guided biopsies or from small-gauge needle biopsies (also referred to as *fine-needle aspirates*), such as those performed during bronchoscopic transbronchial needle aspirations (TBNA) of mediastinal lymph nodes. Therefore, as in our case, ancillary testing is difficult or impossible if the sparse material from small biopsy specimens has already been exhausted for routine diagnostic purposes.

Notwithstanding this, it is to be emphasized the prospective nature of the study adopting liquid biopsy and enrolling consecutive patients with metastatic lung adenocarcinoma, which formed a homogeneous population treated with Pembrolizumab-base systemic therapy. Also, we planned the study focusing on a blood test that could represent dynamic alterations of the evolving cancer. In conclusion, our findings emphasize the possibility of identifying markers associated with different efficacy of immune checkpoint inhibitors in lung cancer. In particular, in the present study, the *KRAS4A* isoform was found to be a promising candidate. Progress along this approach is critical to build reasoning for novel therapeutic combinations and setting a more personalized cancer immunotherapeutic strategy.

## Methods

### Patients and treatments

This is a prospective study, which was performed among three participating Institutions in “Regione Marche” between August 2021 and December 2022. Patients with metastatic lung adenocarcinoma without driver mutations, candidates for first-line systemic therapy, were considered eligible for study entry. Patients with central nervous system metastases were not excluded, provided that they underwent stereotactic radiotherapy of a single lesion documented by computed tomography and magnetic resonance. According to current clinical practice, patients with PD-L1 expression ≥ 50% received Pembrolizumab, whereas patients with PD-L1 expression < 50% received Pembrolizumab and chemotherapy. All patients received 200 mg of Pembrolizumab administered intravenously every three weeks. Chemotherapy consisted of Cisplatin (75 mg per square meter of body-surface area) or Carboplatin (area under the concentration–time curve, 5 mg per milliliter per minute) plus Pemetrexed (500 mg per square meter), all administered intravenously every three weeks. All patients received premedication with folic acid, vitamin B_12_, and glucocorticoids, administered according to local guidelines for pemetrexed use.

Enrolled patients underwent a pre-treatment blood sampling for liquid biopsy analyses. Re-staging procedures were scheduled every twelve weeks or earlier in the case of signs/symptoms of progression^29^. The Immune-modified response evaluation criteria in solid tumors (imRECIST) were used for the assessment of response^30^.

### Real-time quantitative polymerase chain reaction (qPCR)

Samples were collected from the three Oncology Units of the Azienda Sanitaria Territoriale (AST) Pesaro-Fano-Urbino, Regione Marche, Italy. For each patient, two tubes/K_3_EDTA (6 ml each) of fresh peripheral blood were collected. Then, samples were immediately centrifuged at 1830 g for 10 min at 4 °C, and plasma was transferred to a new tube and then stored at − 80 °C. Total RNA was extracted using Trizol LS Reagent (Thermo Fisher Scientific, Waltham, MA, USA) according to the manufacturer's instructions with minor changes. Briefly, 1 volume of plasma was mixed with 3 volumes of TRIzol LS reagent. After 10 min of incubation, for each 750 µl of TRizol used, 200 µl of chloroform was added to the mixture. The mixture was incubated for 3 min at RT and then centrifuged at 3200 g for 75 min at 4 °C. The aqueous layer was transferred to a new tube and measured. RNA precipitation was performed by adding to the aqueous phase 0.8 volumes of isopropanol and 1 μl of GlycoBlue (Thermo Fisher Scientific, Waltham, MA, USA) for each 250 µl of plasma collected. The mixture was left at − 20 °C for 15 min and then centrifuged at 12,000 g for 10 min at 4 °C. The RNA was washed with cold 70% ethanol and air dried for 10 min. Next, 90 μl of DEPC-treated water was added to the pellet and incubated at 56 °C for 10 min. The RNA was quantified at the Qubit™ Fluorometer using Qubit™ RNA HS Assay Kit (Thermo Fisher Scientific, Waltham, MA, USA). Qubit™ Fluorometer uses fluorescent dyes that specifically bind to the target molecule and can distinguish intact from degraded RNA, even in extremely small amounts, providing accurate, specific and sensitive quantification of RNA. In order to overcome the problem of finding a reliable reference gene in the quantitative evaluation of target genes, we chose a different approach, based on the quantification of the RNA by the Qubit™ Fluorometer first, and then adding the same amount of cDNA (7 ng) in each assay. The RNA was concentrated to a final volume of 14 μl and SuperScript™ VILO™ cDNA Synthesis Kit (Thermo Fisher Scientific, Waltham, MA, USA) was used to generate first-strand cDNA according to the manufacturer instructions. The qPCR for the expression analyses of *PD-L1*, *KRAS4A* and *KRAS4B* cmRNA were performed in duplicate. TaqMan probes of the target genes were labeled with two fluorescent dyes that emit at different wavelengths (FAM or VIC for the reporter dye). The following TaqMan Gene Expression Assays, selected to avoid binding to genomic DNA specifically, were chosen: Hs00204257_m1 (*PD-L1*, labeled FAM), Hs00932330_m1 (*KRAS4A*, labeled FAM) and Hs00270666_m1 (*KRAS4B*, labeled VIC) (Thermo Fisher Scientific, Waltham, MA, USA). We carried out co-amplification assays when the probes were labeled with fluorescent dyes that emitted at different wavelengths.

The qPCR reaction volume included 7 ng of the sample, 1 µl of 20X TaqMan Gene Expression Assay of each target, 10 µl of 2 × Hot-Rescue Real-Time PCR Kit-Fluoprobe (MBK0012, Diatheva, Italy), 0.125 µl of Taq Polymerase (5U/µl) (Diatheva, Italy) and H_2_O up to the final volume of 20 µl. The reaction mixture was incubated at 50 °C for 2 min followed by 95 °C for 10 min, and then amplified for 40 cycles at 95 °C for 15 s and 60 °C for 1 min. The qPCR was performed by using an Applied Biosystems 7500 Real-Time PCR system. The cycle-threshold (Ct)-values obtained by the qPCR assays were transformed through an algorithm^31^ into a more accurate and precise value named Cy_0_.

Cy_0_-values were used in subsequent analyses, and they are inversely correlated with the amount of template in the assay. Therefore, a higher cmRNA expression level corresponds to a smaller Cy_0_-value.

### Statistical analysis

The primary end-point of the study was the analysis of the possible association between *KRAS4A* and *KRAS4B* cmRNA expression levels with PFS, defined as the time from the date of the first cycle of therapy to disease progression or death, whichever happened first. Secondary end-points were the association of *KRAS4A* and *KRAS4B* cmRNA expression levels with tumor response and the association of the *KRAS4A* and *KRAS4B* cmRNA expression with *PD-L1* cmRNA levels.

Receiver operating characteristic (ROC) curves served to calculate the Youden index to define the optimum cut-off values for Cy_0_-values for the binary classification of patients^32^.

With 29 events and assuming a 33% prevalence of patients with high *KRAS4A* and high *KRAS4B* cmRNA levels (categorized low Cy_0_-values according to the Youden index), the scenario for sample size estimation would allow the detection of a 66% reduced risk of progression with a power of 80% and a two-sided type I error of 5%. The hypothesized high performance of the studied biomarkers would pursue a meaningful clinical impact, which could justify additional validation studies^33^.

Mean values with 95% confidence interval (CI) for Cy_0_-values were reported and comparisons of means were performed adopting the *t*-test and the Wilcoxon test. The Pearson correlation coefficient was used to determine the correlation between *KRAS4A, KRAS4B* and *PD-L1* cmRNA expression levels. The Fisher’s exact test analyzed categorical variables in contingency tables. The Kaplan–Meier method was used to estimate survival curves and the log-rank test was used to compare survival times between groups. A multivariable Cox proportional hazards model was then used to adjust according to clinical and pathologic features. All reported *p-*values were two-sided and confidence intervals (CIs) were at the 95% level. A *p-*value < 0.05 was considered statistically significant. Significant associations were required to be detectable with the target genes. Analyses were performed using MedCalc for Windows, version 15.0 (MedCalc Software, Ostend, Belgium).

### Ethical approval

This was a local study, involving three hospitals: Pesaro, Fano and Urbino of Marche Region (Italy). For this reason it was not registered in the clinicaltrials.gov registry. The study was performed in accordance with the International Conference on Harmonization Good Clinical Practice Guidelines, the Declaration of Helsinki (1996) and approved by the local Ethics Committee (CERM, Comitato Etico Regionale delle Marche) on 29/07/2021 (Protocol code n. 2021 234). The datasets generated and/or analyzed during the current study are not publicly available due indications by the ethical committee, but they are available from the corresponding author on reasonable request.

### Informed consent

All patients gave written informed consent before being included in the study.

## Data Availability

Research data supporting this publication are available upon request from the corresponding author.
